# Translation of the 5D Itching Scale from English to Malay, and Its Validation among Patients with Chronic Kidney Disease in Malaysia

**DOI:** 10.3389/fmed.2017.00189

**Published:** 2017-11-08

**Authors:** Inayat Ur Rehman, David Bin-Chia Wu, Siew Mei Pauline Lai, Uma Devi Palanisamy, Soo Kun Lim, Tahir Mehmood Khan

**Affiliations:** ^1^School of Pharmacy, Monash University, Bandar Sunway, Malaysia; ^2^Department of Primary Care Medicine, University of Malaya, Kuala Lumpur, Malaysia; ^3^Jeffrey Cheah School of Medicine and Health Sciences, Monash University, Bandar Sunway, Malaysia; ^4^Department of Nephrology, University of Malaya, Kuala Lumpur, Malaysia

**Keywords:** pruritus, chronic kidney disease, 5D itching scale, Malay, validity, reliability

## Abstract

**Introduction:**

Several tools have been developed to assess the severity of pruritus. In Malaysia, no tool has been validated to assess pruritus in patients with chronic kidney disease (CKD). Therefore, the aim of our study was to validate the Malay 5D itching scale (M5D-IS) among patients with CKD in Malaysia.

**Method:**

The English version of the 5D-IS was translated into Malay according to International Guidelines. Face and content validity was determined by an expert panel and pilot tested in patients with end-stage renal disease (ESRD). The M5D-IS was then validated in a tertiary hospital in Malaysia from May to June 2016. We recruited patients with (i.e., patients with ESRD) and without pruritus (i.e., patients with stage 1–3 CKD) (to determine if the M5D-IS could discriminate between the two groups), and administered the M5D-IS at baseline and 2 weeks later. Exploratory factor analysis was used to examine the construct validity. Internal consistency was assessed using Cronbach’s alpha and intraclass correlation coefficient was calculated to assess the reliability of the instrument.

**Results:**

A total of 70 participants were recruited (response rate = 100%). The majority were males (51.4%) and Malay (67.1%). Exploratory factor analysis revealed that the 5D-IS had 2-factor loadings: “daily routine activity” and “pattern of itching,” which explained 77.7% of the variance. The overall score of the M5D-IS, as well as for each domain, was significantly worse in participants with pruritus (9.83 ± 0.35), compared to those without pruritus (5.51 ± 0.93, *p* < 0.001). The overall Cronbach’s alpha for the M5D-IS was (0.861), indicating adequate internal consistency. At test–retest, the intraclass correlation coefficient was significantly correlated.

**Conclusion:**

The M5D-IS was found to be a valid and reliable instrument to assess pruritus among patients with CKD in Malaysia.

## Introduction

Worldwide, the incidence of chronic kidney disease (CKD) has increased significantly during the past decades (1, 2). In 2013, approximately 956,200 deaths were due to CKD, which was an increase of 134.6% from 1990 (3). CKD is ranked as the 27th and 19th leading disease based on its mortality in 2010 (4) and 2013 (5), respectively. In Malaysia, there were 32,026 patients on dialysis in the year 2014; of which, 29,192 (91%) were on hemodialysis and 2,834 (9%) were on peritoneal dialysis (6). By year 2040, it has been estimated that the number of patients with end-stage renal disease (ESRD) would triple from existing 2014 figures (7).

Pruritus is a common complication in patients with CKD (8–10). Pruritus usually develops about 6 months after the initiation of dialysis and affects approximately 50–90% of patients (11–14). The symptoms of pruritus range from mild to severe and can be caused by dermatological (e.g., xerosis) or systemic causes (e.g., uremia) (15). Pruritus may present as either an acute or chronic condition (11), be generalized, or localized; and may last from a few months to more than 1 year (16). The most affected parts are the face, back, and fistula arm (17). Pruritus affects the quality of life of patients (11, 18–21), as it compromises physical and mental wellbeing (22, 23). In Malaysia, pruritus affects approximately 60% of patients undergoing dialysis (24, 25).

To assess the severity of pruritus, several instruments have been developed: the 5D itching scale (5D-IS) (26), the Visual Analog Scale (20), the Eppendorf Itch Questionnaire (27), and the Skindex-10 (28). Among these instruments, the 5D-IS developed by Elman et al. (26), which consists of 5 domains with 8 items, is the most promising instrument, as it has been specifically designed to assess pruritus in patients suffering from liver/renal disease or burns. The 5D-IS is a self-administered tool that assesses the impact of itch on the quality of life and is able to detect changes over time (26). However, the 5D-IS has not been validated in Malaysia. Malay is a major language of the Austronesian family, the national language of Malaysia, Brunei, and Indonesia, and spoken by 270 million people (29). Therefore, the aim of this study was to translate the English version of the 5D-IS from English to Malay and to validate the Malay 5D-itching scale (M5D-IS) among patients with CKD in Malaysia.

## Materials and Methods

This validation study was performed from May to June 2016 at a tertiary hospital in Kuala Lumpur, Malaysia.

### Participants

To assess the discriminative validity of the M5D-IS, we recruited patients that had pruritus (i.e., patients with ESRD) and those that did not (i.e., patients with stage 1–3 CKD). We hypothesized that patients with pruritus would have a worse quality of life (i.e., a higher M5D-IS score) than patients without pruritus. Patients aged 21 years and above who could answer the questionnaire in Malay and undergoing dialysis were recruited from the dialysis unit; while those with stages 1–3 CKD were recruited from the Nephrology clinic. Excluded were those who had pancreatitis, liver complications (e.g., hepatitis A, B, or C), or an autoimmune disease (e.g., systemic lupus erythematous).

### Sample Size

Sample size was calculated based on the number of items to participant ratio of 1:10 required to perform this validation study (30). The M5D-IS consists of eight items, of which, seven items were measured on a five-point Likert scale, and one item on a nominal scale. Hence, only seven items could be validated, and the minimum sample size required was 7 × 10 = 70 participants.

### Instruments Used

#### The M5D-IS

**Figure 1:**
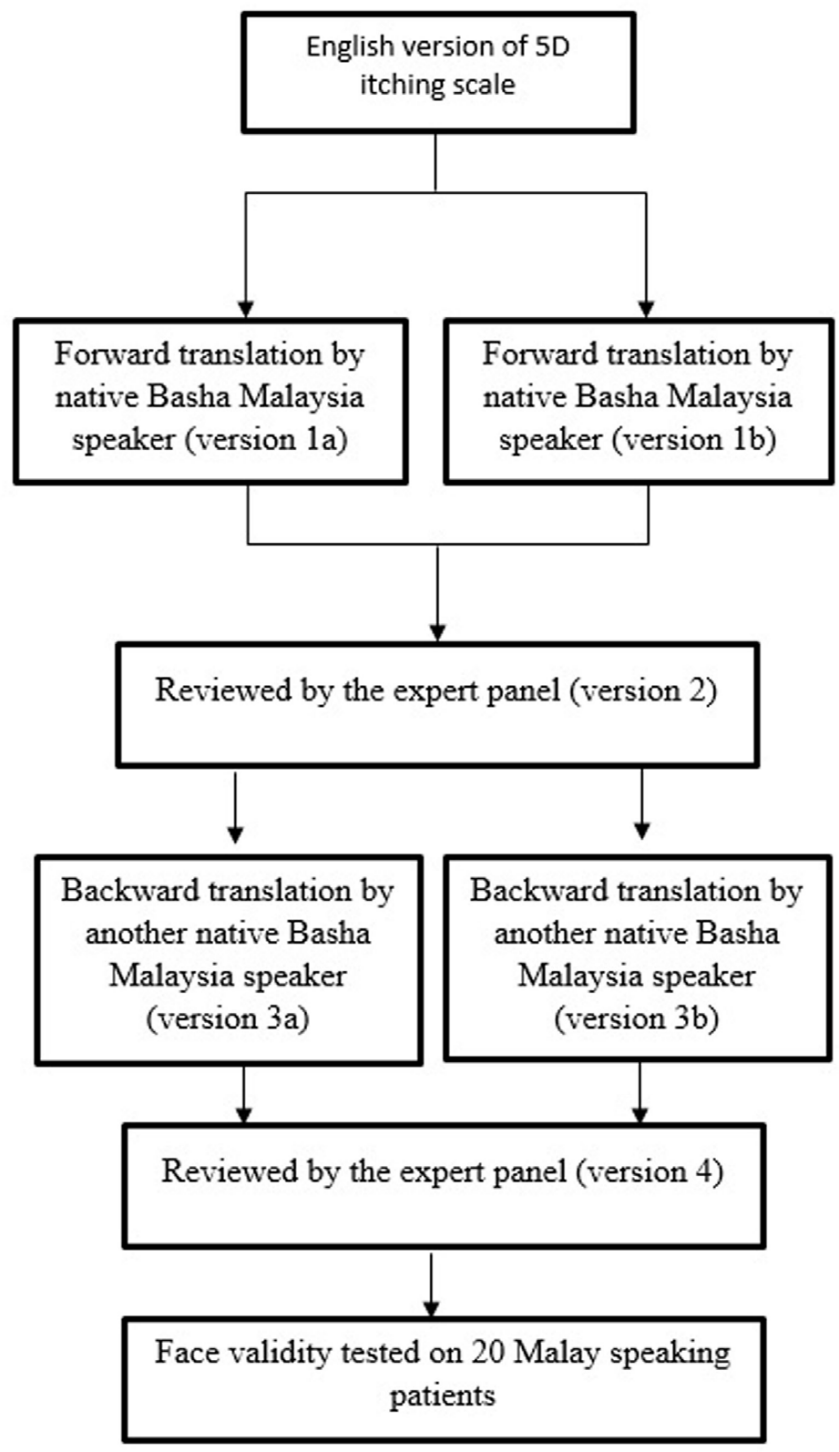
Translation of the 5D itching scale from English to Malay.

The M5D-IS was originally developed in English by Elman et al. (26). It consists of eight items with five domains, addressing the duration, degree, direction, disability, and distribution of itching. The 5D-IS was translated from English to Malay according to International Guidelines (31, 32) (Figure 1; Datasheet S1 in Supplementary Material).

The duration, degree, and direction domains each included one item, while the disability domain had four items. All items of the first four domains were measured on a five-point Likert scale (where 1 represented the lowest degree of pruritus and 5 represented the highest degree). The fourth domain (disability) assessed the effect of itching on daily activities, and its score was calculated by selecting the highest score. In the fifth domain, participants were asked to select which part of the body was most affected by pruritus, and participants could select as many parts as they wished. If two body parts were affected, the score given was 1; 3–5 body parts affected was scored as 2, 6–10 body parts were scored as 3, 11–13 body parts were scored as 4, and 14–16 body parts were scored as 5. The overall score of the M5D-IS was calculated by summing all the five domains; 5 indicates no pruritus; while a score of 25 indicates severe pruritus (17).

### Procedure

Patients were approached while they were undergoing dialysis or waiting to see the doctor in the nephrology clinic. The purpose of the study was explained to them. For those who agreed to participate, written informed consent was obtained. Participants were asked to fill in the baseline demographic form and the M5D-IS. To assess for reliability, the M5D-IS was administered to the same participants 2 weeks later.

### Statistical Analysis

Data were analyzed using the Statistical Package for Social Sciences version 20.0 (SPSS Inc., Chicago, IL, USA). Normality was assessed using the Shapiro–Wilks test (33). Since our data were not normally distributed, non-parametric tests were used. Continuous variables were presented as median and interquartile range, while categorical variables were presented as number and frequency.

### Validity

Exploratory factor analysis was conducted using principal component analysis and varimax rotation. Items were then screened to identify those with factor loading >0.4 (34). Sampling adequacy for factor analysis was assessed using Kaiser–Mayer–Olkin (KMO), factor loadings, average variance extracted (AVE), and composite reliability (CR), where values greater of more than 0.7, 0.5, 0.5, and 0.6, respectively, indicate a good model of fit (35).

### Discriminative Validity

The Mann–Whitney *U*-test was used to determine whether the M5D-IS was able to discriminate between patients with and without pruritus. The significance level was set at *p* < 0.05.

### Reliability

Cronbach’s alpha was used to assess the internal consistency of the M5D-IS. A Cronbach’s alpha value >0.70 indicates good internal consistency (36). Corrected item-total correlation (CITC) should be >0.4 to be considered acceptable (37). The Cronbach alpha value was also computed if an item was deleted. Test–retest was assessed using the intraclass correlation coefficient (ICC) to examine the agreement between the repetitive measures. The ICC values greater than 0.75 indicates excellent agreement, from 0.60–0.74 indicates good agreement; 0.40–0.59 shows fair to moderate agreement, and less than 0.40 indicates poor agreement (38).

## Results

A total of 70 participants were approached, and all agreed to participate (response rate = 100%). The majority were males (51.4%), and Malay (67.1%), with a median age of 58.3 (IQR = 15) years. A total of 45.7% only finished secondary level education. Medical treatment was borne mainly by the government (61.4%) and family (34.3%). The majority of our participants were financially dependent on family (41.4%) and the majority (27.1%) only had a monthly income of RM 1,000–2,000 (USD$245–491) (Table 1).

**Table 1:** Demographic characteristics of participants (*n* = 70).

Demographic characteristics	*N* (%)
**Gender**	
Male	36 (51.4)
Female	34 (48.6)
**Ethnicity**	
Malay	47 (67.1)
Chinese	15 (21.4)
Indian	8 (11.4)
**Median age (years) (IQR)**	
Median	58.3 years
IQR	(15)
**Education**	
Primary level (6 years of education)	14 (20)
Secondary level (12 years of education)	32 (45.7)
College/diploma (15 years of education)	13 (18.5)
Tertiary (≥16 years of education)	11 (15.7)
**Marital status**	
Single	4 (5.7)
Married	66 (94.3)
**Job status**	
Working	12 (17.2)
Not working	58 (82.8)
Dependent on family	30 (42.9)
**Expenses for treatment borne by**	
Government	43 (61.4)
Family	24 (34.3)
Employer	2 (2.9)
Self	1 (1.4)
**Monthly income**	
Dependent on family	29 (41.4)
RM 1,000–2,000 (USD$245–491)	19 (27.1)
RM 2,000–3,000 (USD$491–736 USD)	9 (12.9)
RM 4,000–5,000 (USD$982–1,227 USD)	6 (8.6)
RM 5,000–60,000 (USD$1,227–1,473 USD)	2 (2.9)
RM 6,000 and above (USD$1,473 USD and above)	5 (7.1)

### Validity

Exploratory factor analysis revealed that the M5D-IS had two factor loadings: “daily routine activity” and “pattern of itching” (Table 2). This explained 77.7% of the variance. The overall score of the M5D-IS, as well as for each domain, was significantly worse in participants with pruritus (9.83 ± 0.35), compared to those without pruritus (5.51 ± 0.93, *p* < 0.001) (Table 3).

**Table 2:** Exploratory factor analysis of the Malay 5D-itch scale.

Domains	Factor 1	Factor 2	KMO	CR	AVE
	Daily routine activity	Pattern of itching			
Duration		0.70	0.78	0.84	0.63
Degree		0.87			
Direction		0.76			
Sleep		0.83			
Leisure/social	0.95		0.71	0.94	0.87
House work	0.93				
Work/school	0.87				

*Extraction method: principal component analysis. Rotated method: varimax*.

*KMO, Kaiser–Mayer–Olkin; CR, composite reliability; AVE, average variance extracted*.

**Table 3:** Score of chronic kidney disease (CKD) stage 1–3 and end-stage renal disease (ESRD) patients with pruritus or without pruritus for Malay 5D itch scale (M 5D-IS).

Domains	Patients with ESRD (*n* = 35)		Patients with stage 1–3 CKD (*n* = 35)		Mann–Whitney *U*-test	
		
	Median	Mean ± SD	Median	Mean ± SD	*z*-score	*p*-Value
Duration	1.00	1.11 ± 0.55	1.00	0.89 ± 0.18	−6.86	<0.001*
Degree	3.00	3.03 ± 0.11	1.00	1.23 ± 0.21	−6.09	<0.001*
Direction	2.00	2.63 ± 0.21	1.00	1.29 ± 0.07	−6.05	<0.001*
Disability						
Sleep	1.00	1.26 ± 0.07	1.00	0.77 ± 0.17	−6.86	<0.001*
Leisure/social	1.00	1.14 ± 0.10	1.00	1.26 ± 0.28	−7.03	<0.001*
House work	1.00	1.20 ± 0.12	1.00	1.23 ± 0.28	−6.94	<0.001*
Work/school	1.00	1.00 ± 0.24	1.00	1.00 ± 0.24	−7.60	<0.001*
Distribution	1.00	1.54 ± 1.32	1.00	0.69 ± 0.11	−7.50	<0.001*
Overall score	10.00	9.83 ± 0.35	5.00	5.51 ± 0.93	−5.96	<0.001*

*Statistically significant at p < 0.05.

### Reliability

The overall Cronbach’s alpha for the Malay 5D-IS was 0.861. At retest, six participants were lost to follow-up. The intraclass correlation coefficient (ICC) at test retest for all domains were statistically significant (*p* < 0.001) and showed excellent agreement for all items except for the items on sleep and distribution (which showed good agreement) (Table 4).

**Table 4:** The psychometric properties of the Malay 5D-itching scale.

Domain	Cronbach’s alpha for overall instrument	Corrected item total correlation	Cronbach’s alpha if item deleted	Test (*n* = 70)	Retest (*n* = 64)	Intra class correlation coefficient (ICC)*
				Median	Median	
Duration	0.861	0.62	0.84	1.00	1.00	0.94*
Degree		0.64	0.84	3.00	2.00	0.93*
Direction		0.65	0.84	2.00	2.00	0.91*
Disability						
Sleep		0.86	0.86	1.00	1.00	0.69*
Leisure/social life		0.69	0.83	1.00	1.00	0.97*
House work		0.71	0.82	1.00	1.00	0.97*
Work/school		0.68	0.83	1.00	1.00	0.82*
Distribution		0.76	-	1.00	1.00	0.61*

*Statistically significant at p < 0.05; thighs, lower leg, and forearm are the most affected body parts reported by respondents.

## Discussion

The M5D-IS was found to be a reliable and valid tool to assess pruritus among patients with CKD. The M5D-IS was found to be a 2-factor model, was able to discriminate between participants with and without pruritus, and had adequate psychometric properties.

EFA found that the M5D-IS was a 2-factor model. The first factor—“pattern of itch” consists of four domains: degree, duration, direction, and sleep; while the second factor was on “daily routine activity.” EFA found that “sleep” fit items that assessed the degree, duration, and direction of pruritus, instead of “daily routine activity” (which was part of the original instrument). This may be because sleep disturbance due to pruritus is a common occurrence, and is dependent on the degree, duration, and direction of pruritus (12, 39). The factor on “daily routine activity” had three items, i.e., leisure/social, house work, and work/school. These activities were disturbed by the presence of pruritus, but did not have a major impact on the quality of life. Previous studies (15, 26) did not perform exploratory factor analysis. Hence, we are unable to compare our results. The M5DIS score was significantly different between patients with and without pruritus, indicating that our instrument was able to discriminate between these two groups. We were not able to compare our results with previous studies as discriminative validity was not assessed.

The internal consistency of the M5D-IS was >0.7, which was similar to previous validation studies (15, 26, 40, 41). This indicates that our instrument has achieved adequate internal consistency. For test–retest, the ICC values showed good and excellent agreement. Our ICC value was lower than a previous study. This could be because we assessed the reliability of our instrument 14 days after baseline while the other study assessed reliability 2 days after baseline (26). Pruritus in our participants could have improved after 2 weeks, thereby lowering the ICC value in our study. Nevertheless, our instrument still demonstrated adequate reliability.

The strength of our study was that we recruited sufficient participants to perform factor analysis and discriminative validity. However, we were not able to perform convergent validity, as there were no other validated instrument available in Malay to assess pruritus during the period of our study. In addition, we only recruited patients from one center using convenience sampling. Hence, our results are not generalizable.

## Conclusion

The M5D-IS was found to be a valid and reliable instrument for assessing pruritus among patients with CKD in Malaysia. Future studies should validate the tool in English and Mandarin so that this tool can be administered to Malaysians that can only understand these languages.

## Ethics Statement

Monash University Human Research and Ethics Committee (MUHREC Approval No: CF16/1766-2016000890) and the University Malaya Medical Centre Medical Ethics Committee approved this study (Approval No: 20163-2303).

## Author Contributions

Conceived and design: IR and TK. Data collection: IR, LK, and SL. Analyzed the data: IR, SL, and TK. Interpretation of data/result: IR, TK, DW, and SL. Read and approved the final version: TK, SL, and UP. All authors approved the submission of the final manuscript.

## Conflict of Interest Statement

The authors declare that the research was conducted in the absence of any commercial or financial relationships that could be construed as a potential conflict of interest.

## Abbreviations

5D-IS, 5D itching scale; CKD, chronic kidney disease; VAS, visual analog scale; EIQ, Eppendorf Itch Questionnaire; MUHREC, Monash University Human Research and Ethics Committee; ICC, intraclass correlation coefficient; KMO, Kaiser–Mayer–Olkin.
