# Supplementary material for: Translation of the 5D Itching Scale from English to Malay, and Its Validation among Patients with Chronic Kidney Disease in Malaysia
Source: Front Med (Lausanne). 2017 Nov 8;4:189. doi: 10.3389/fmed.2017.00189 (PMC5682308; doi:10.3389/fmed.2017.00189)
Supplement: Supplementary file 1 [file Data_Sheet_1.PDF]

Malay 5D itch scale (M5D-IS)

Kami menghargai dan mengucapkan terima kasih atas kerjasama anda dalam memberikan input untuk menjawab soal selidik ini. Penemuan daripada kajian ini akan dirahsiakan untuk tujuan penyelidikan sahaja.

1. **Jangka masa:** Dalam masa 2 minggu lepas, dalam berapa jam semasa yang anda rasa kulit kegatalan

Kurang daripada 6 jam /hari	6-12 jam sehari	12-18 jam sehari	18-23 jam sehari	Sepanjang hari
☐	☐	☐	☐	☐
1	2	3	4	5

2. **Tahap kegatalan:** Sila beri tahap kegatalan kulit dalam masa 2 minggu lepas

Tidak	hadir Ringan	Sederhana	teruk	Unbearable
☐	☐	☐	☐	☐
1	2	3	4	5

3. **Penambahbaikan:** Dalam masa 2 minggu lepas, adakah kegatalan kulit anda menjadi lebih baik atau **lebih** teruk jika dibandingkan dengan bulan lepas

sepenuhnya Diselesaikan	Lebih baik, tetapi masih ada	Sedikit lebih baik, etapi masih ada	Tidak berubah	Semakin teruk
☐	☐	☐	☐	☐
1	2	3	4	5

4. **Kurang upaya:** Berikan tahap kesan kegatalan atas aktiviti berikut dalam masa 2 minggu lepas

Tidur

Tidak ganggu Tidur	Ada kala ganggu masuk tidur	Selalu ganggu masuk tidur	Ganggu masuk tidur dan ada kala mengejutkan masa malam	Ganggu masuk tidur dan selalu mengejutkan masa malam
☐	☐	☐	☐	☐
1	2	3	4	5

	N/A	Jarang melaksanakan aktiviti	Jarang aktiviti kesan	Kadang-kadang kesan aktiviti	Kerap melaksanakan aktiviti	Sentiasa melaksanakan aktiviti
**Aktiviti harian**	☐	☐ 1	☐ 2	☐ 3	☐ 4	☐ 5
**Kerja-kerja rumah**	☐	☐ 1	☐ 2	☐ 3	☐ 4	☐ 5
**Kerja / Sekolah**	☐	☐ 1	☐ 2	☐ 3	☐ 4	☐ 5

5. **Tempat mengalami kegatalan:** Tandakan kawasan badan yang anda rasa kegatalan dalam 2 minggu lepas.

	Hadir		Hadir
Ketua / kulit kepala	☐	Kaki tunggal	☐
Muka	☐	Tapak tangan	☐
Dada	☐	Jari tangan	☐
Abdomen	☐	Lengan	☐
Kembali	☐	Lengan Atas	☐
Punggung	☐	Tempat Hubungi w / Pakaian (Contohnya tali pinggang, pakaian)	☐
Paha	☐	Pangkal paha	☐
kaki Lowe	☐	Puncak kaki / kaki	☐
